# Deciphering the Cellular Effects of Strontium Chloride and Potassium Carbonate on Induced Pluripotent Stem Cells and Their Derivative Cardiomyocytes

**DOI:** 10.3390/ph19030362

**Published:** 2026-02-25

**Authors:** Saheera Kumar, Michelle Vanessa Kamga Kapchoup, Hai Zhang, Sureshkumar Perumal Srinivasan, Adeline Kaptue Wuyt, Jude Tsafack Zefack, Jürgen Hescheler, Filomain Nguemo

**Affiliations:** 1 Centre for Physiology, Faculty of Medicine and University Hospital Cologne, University of Cologne, 50931 Cologne, Germanymichelle.kamga@thermofisher.com (M.V.K.K.); sureshps_bio@yahoo.com (S.P.S.); j.hescheler@uni-koeln.de (J.H.); 2 Thermo Fischer Scientific, Baden-Württemberg, 76227 Karlsruhe, Germany; 3 State Key Laboratory of Southwestern Chinese Medicine Resources, College of Pharmacy, Chengdu University of Traditional Chinese Medicine, Chengdu 611137, China; zhanghai@cdutcm.edu.cn; 4 Research Unit of Animal Physiology and Phytopharmacology, Faculty of Sciences, University of Dschang, Dschang P.O. Box 67, Cameroon; adeline.wuyt@yahoo.fr; 5 Engelhardt School of Global Health and Bioethics, Euclid University, Bangui, Central African Republic; judetsafackz@gmail.com

**Keywords:** dental, strontium chloride, potassium carbonate, cytotoxicity, pluripotent stem cells, proliferation

## Abstract

**Background/Objectives**: Toothpaste ingredients such as strontium chloride (SrCl_2_) and potassium carbonate (K_2_CO_3_) are recognized for their desensitizing and remineralizing effects but may be absorbed through the oral mucosa. Their potential cytotoxic and cardiotoxic properties, however, remain inadequately characterized. Here, we investigated the effects of SrCl_2_ and K_2_CO_3_ on mouse-induced pluripotent stem cells (iPSCs) and iPSC-derived cardiomyocytes (iPSC-CMs). **Methods:** Cells were exposed to varying concentrations of each compound for up to 72 h. Real-time cell analysis (xCELLigence RTCA Cardio system) was used to assess proliferation, and flow cytometry was used to evaluate cell viability. Functional properties of iPSC-CMs were examined using multi-electrode array (MEA) recordings and xCELLigence-based impedance measurements. Cardiac marker expression was examined via immunofluorescence and quantitative RT-PCR. **Results:** Both SrCl_2_ and K_2_CO_3_ affected iPSC proliferation and reduced viability in a dose- and time-dependent manner, accompanied by altered embryoid body (EB) morphology and increased cell death. In iPSC-CMs, both compounds downregulated key cardiac genes and disrupted spontaneous beating activity, with effects intensifying at higher concentrations. **Conclusions:** These results demonstrate that SrCl_2_ and K_2_CO_3_ induced dose-dependent cytotoxic and arrhythmogenic effects on iPSCs and iPSC-CMs. At elevated concentrations, these compounds impair iPSC-CM function and may pose safety concerns upon chronic exposure. Further mechanistic and long-term in vivo studies are warranted to assess their potential cardiotoxic risk in consumer oral care products.

## 1. Introduction

Toothpaste is an essential component of daily oral hygiene and is widely used across all age groups. Although not intended for ingestion, many of its components can remain in the oral cavity after brushing. The oral mucosa, due to its high permeability and vascularization, enables rapid absorption of substances [1]. Consequently, ingredients in toothpaste may exert effects beyond the oral cavity, potentially influencing sensitive organs such as the heart. Modern toothpaste formulations contain a range of active and inactive ingredients, such as abrasives, humectants, surfactants, flavoring agents, and preservatives, along with pharmacologically active components targeting specific oral health issues such as tooth sensitivity [2,3]. SrCl_2_ is a commonly used desensitizing agent that acts by occluding dentinal tubules, thereby reducing fluid movement and neural excitation [4,5]. Beyond its role in dentistry, SrCl_2_ has been shown to alleviate skin irritation [6,7], and to promote periodontal cell proliferation and mineralization at low concentrations [8,9]. Despite these beneficial effects, concerns have been raised about its safety, particularly with regard to cardiovascular health. Epidemiological studies have linked strontium-based medications to an increased risk of cardiovascular events [10], although the exact underlying mechanisms remain unclear. Importantly, the direct effects of SrCl_2_ on iPSCs and iPSC-CMs have not been investigated.

Potassium carbonate (K_2_CO_3_) is another ingredient occasionally included in natural or mild toothpaste formulations, though its specific role in these products remains poorly defined. K_2_CO_3_ is widely used in industrial and pharmaceutical contexts [11,12,13]. However, due to its alkaline nature, K_2_CO_3_ may act as a mucosal irritant at high concentrations, suggesting possible biological reactivity [13]. Its effects on excitable cells, including CMs, are largely unknown, and data on its cardiac safety are currently lacking.

To address these knowledge gaps, the present study investigated the impact of these two widely used toothpaste ingredients, SrCl_2_ and K_2_CO_3_, on cell viability, electrophysiological function, and cardiac gene expression in a murine iPSC-based in vitro model. Cell proliferation and viability were assessed in undifferentiated iPSCs, while functional and molecular endpoints were evaluated in iPSC-CMs. A combination of advanced and innovative analytical tools and techniques, including real-time cell analysis (xCELLigence RTCA Cardio system), multi-electrode array (MEA) recordings, Fluorescence-Activated Cell Sorting (FACS), immunocytochemistry, and quantitative RT-PCR, was employed to characterize both acute and dose-dependent effects. By integrating multiple complementary assays within a physiologically relevant system, this study provides novel insights into the toxicological and functional profiles of SrCl_2_ and K_2_CO_3_. The findings may help refine safety assessments for dental care products and guide future research on potential side effects of topically applied compounds.

## 2. Results

### 2.1. Mouse iPSC Maintenance and Cardiac Differentiation

**Figure 1:**
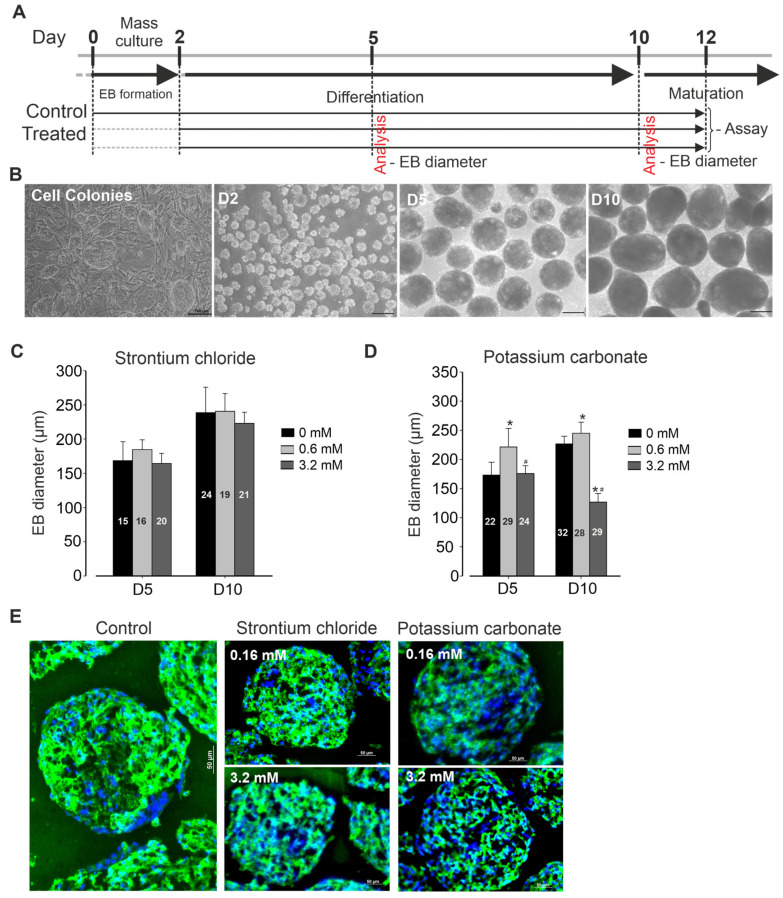
Differentiating murine induced pluripotent stem cells (iPSCs) into cardiomyocytes (CMs). (**A**) Schematic representation of the experimental protocol. (**B**) Representative microscopic images showing cell colonies and embryo bodies (EB) at different stages of differentiation. (**C**,**D**) Bar graphs showing the number of observations (*n*) used for statistical analysis of EB diameters under strontium chloride (**C**) and potassium carbonate (**D**) treatments compared to control conditions. ach bar represents the mean EB diameter, with error bars indicating standard deviation (SD). Graphs illustrate EB size across differentiation stages, highlighting the impact of the treatments on EB growth and development. (**E**) Staining of cardiac α-actinin (green) on non-dissociated clusters of beating-day-14 EBs differentiated in the absence or presence of the indicated compound. Nuclei were counterstained with Hoechst. * *p* < 0.05 vs. control, # *p* < 0.05 vs. previous concentration.

Cell culture protocol is depicted in Figure 1A. Briefly, murine iPSCs (AT25) were stably maintained on a neomycin-resistant feeder layer, exhibiting typical colony morphology during expansion, with passaging occurring every 2 days. Differentiation was initiated through embryoid body (EB) formation (Figure 1B) in the presence or absence of various concentrations of SrCl_2_ and K_2_CO_3_. After two days of suspension culture, EBs were successfully formed. These EBs were then exposed to the respective compound treatments and monitored throughout the subsequent period until final analysis. At days 5 and 10 post-differentiation, no significant changes in EBs size were observed across all concentrations of SrCl_2_ tested, as compared to control conditions (Figure 1C). In contrast, exposure to K_2_CO_3_ at low concentration (0.6 mM) resulted in significant increase in EB size. However, at high concentration (3.2 mM), K_2_CO_3_ induced changes in EB size, decreasing it to approximately half the size observed in the control condition (Figure 1D). Spontaneous beating of the EBs was generally first observed between day 6 and 8 post-differentiation. Simultaneously, eGFP expression under the myosin heavy chain promoter was detected in beating areas, indicating cardiomyogenic lineage commitment. Immunofluorescence analysis of EB clusters revealed positive staining for α-actinin, a cardiac-specific marker. As shown in Figure 1E, expression of α-actinin was less intense in EBs treated with high concentrations of K_2_CO_3_ compared to controls, suggesting that these treatments may at least in part affect cardiomyogenesis.

### 2.2. SrCl_2_ and K_2_CO_3_ Reduce iPSC Proliferation

**Figure 2:**
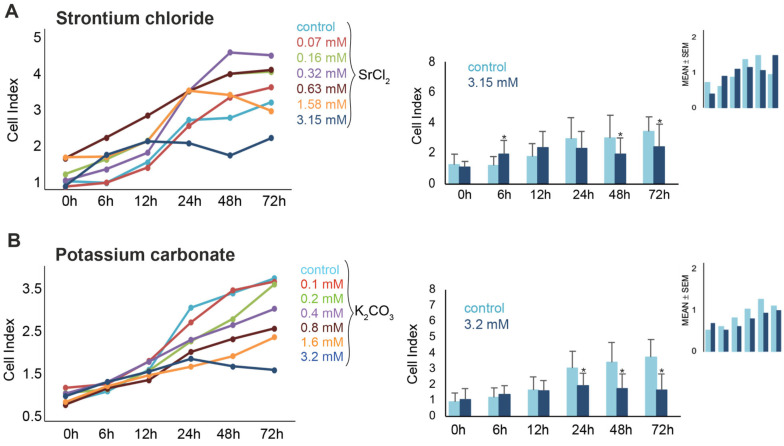
Dose- and time-dependent effects of SrCl_2_ and K_2_CO_3_ on iPSC proliferation measured via xCELLigence real-time cell analysis. (**A**) SrCl_2_ exposure induced a variable response in murine iSPCs. Lower concentrations (≤0.68 mM) showed a moderate increase in cell index (CI) over time, whereas higher concentrations (1.58–3.15 mM) caused a delayed suppression of CI, particularly after 48–72 h (*p* < 0.05). (**B**) K_2_CO_3_ exposure led to a more pronounced and concentration-dependent inhibition of proliferation. CI declined significantly at ≥1.6 mM starting at 24 h (*p* < 0.05). Right bar graphs show mean CI values ± SD at 3.15 mM vs. control over time (*n* = 3). Small insets (top right) illustrate inter-experimental variability for 3.15 mM vs. control, shown as CI range with error bars. These highlight that the observed effects, especially for SrCl_2_, were subject to biological variation across independent experiments. Data are presented as mean ± SD from three independent experiments, each including six wells per condition in a 96-well plate. * *p* < 0.05.

The effects of both compounds, SrCl_2_ and K_2_CO_3,_ on the viability of murine pluripotent stem cells were further examined over a period of 72 h. Real-time impedance monitoring revealed a biphasic response to SrCl_2_ exposure (Figure 2A). At lower concentrations (0.07–0.63 mM), a transient increase in CI was observed during the first 6–12 h. However, higher concentrations (≥1.58 mM) induced a progressive decline in CI, with a significant reduction at 3.15 mM (down to ~46% of control at 72 h, *p* < 0.05). Intermediate concentrations (0.16–0.63 mM) occasionally exhibited proliferative effects depending on the replicate, suggesting inter-experimental variability. Microscopic analysis confirmed reduced cell density and apoptotic morphology after 3.15 mM treatment. FACS further validated this effect, showing a dose-dependent increase in PI-positive cells from 2.4% (control) to 12.0% at 3.15 mM after 72 h (*p* < 0.05).

**Figure 3:**
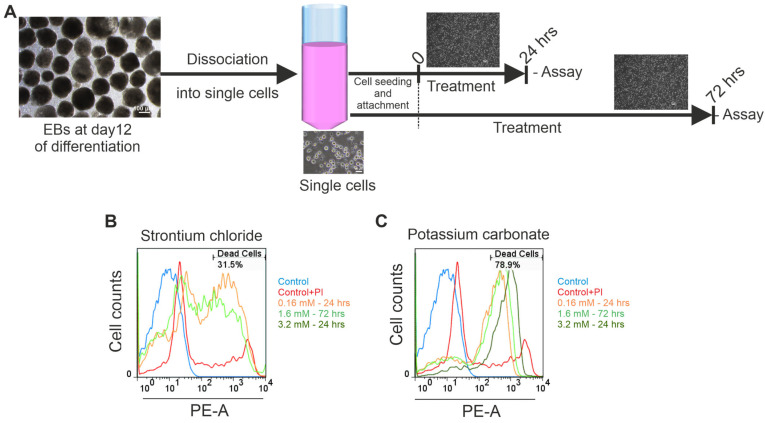
Assessment of cell viability and structural integrity in iPSC-CMs following SrCl_2_ and K_2_CO_3_ exposure. (**A**) Diagram of the experimental protocol for flow cytometry analysis illustrating the EBs’ dissociation, cell seeding, and treatment timeline. (**B**,**C**) Representative flow cytometry plots showing PI-positive cells within the population treated with varying concentrations of SrCl_2_ (**A**) and K_2_CO_3_ (**B**). The plot demonstrates a significant rightward shift in the treated cells, both in a concentration- and time-dependent manner, compared to untreated cells, indicating cell damage. (PI, propidium iodide).

Exposure to K_2_CO_3_ resulted in a reproducible, concentration- and time-dependent decrease in cell viability (Figure 2B). Real-time impedance analysis showed significant CI reductions at 1.6 mM and 3.2 mM K_2_CO_3_, beginning as early as 24 h and persisting through 72 h. At 3.2 mM, CI values dropped below 50% of the control by 48–72 h (*p* < 0.05), indicating pronounced cytotoxicity. In contrast to SrCl_2_, no significant proliferative responses were observed at any concentration of K_2_CO_3_ before 24 h. Microscopy examination of cells treated with 3.2 mM for 72 h revealed reduced cell density, detachment, and morphological signs of cell damage Figure 1C,D). These findings were further corroborated by FACS data (Figure 3), which revealed a significant increase in PI-positive cells to 12.7% at 24 h, and 16.2% at 72 h (both *p* < 0.05 vs. control), confirming the cytotoxic effect. Taken together, these results demonstrate that exposure to K_2_CO_3_ consistently induced dose-dependent iPSC death, with high concentrations leading to substantial loss of viability.

### 2.3. SrCl_2_ and K_2_CO_3_ Modulate Beating Activity of iPSC-CM Clusters Assessed by MEA

**Figure 4:**
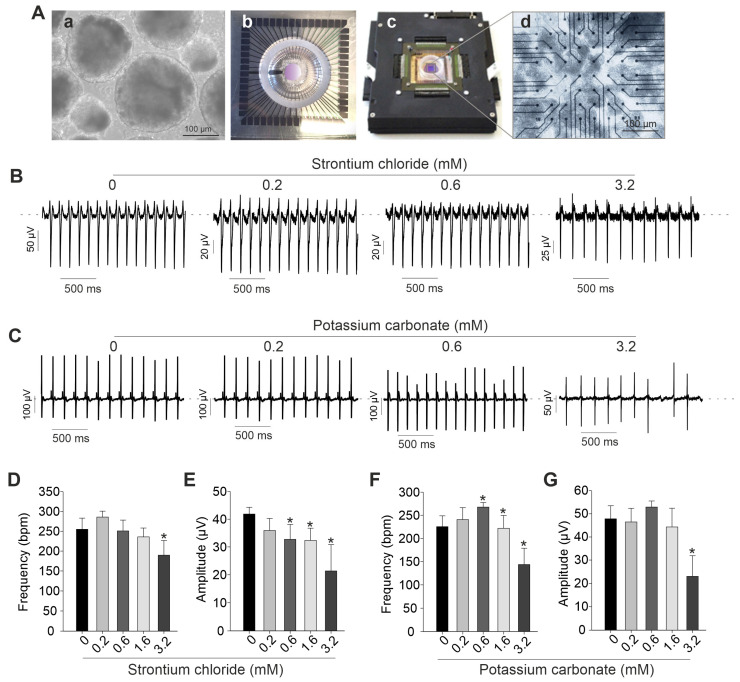
Functional effects of SrCl_2_ and K_2_CO_3_ on iPSC-CM beating clusters were assessed by MEA. (**A**) The setup and cell model used for MEA analysis. (**a**) Brightfield image of EBs at day 12 post-differentiation; (**b**–**d**) MEA chip and amplifier system used for field potential recordings of iPSC-CMs seeded on MEA electrodes. (**B**,**C**) Representative FP traces of iPSC-CMs treated with increasing concentrations of SrCl_2_ (**B**) or K_2_CO_3_ (**C**). Traces demonstrate effects on frequency and FP amplitude across 0–3.2 mM concentrations. (**D**,**E**) Quantification of beating frequency ((**D**), *n* = 4) and FP amplitude ((**E**), *n* = 3) in SrCl_2_-treated iPSC-CMs. While beating frequency was not significantly altered at lower doses, a significant reduction was observed at 3.2 mM. Field potential (FP) amplitude decreased significantly at concentrations ≥ 0.2 mM. (**F**,**G**) Quantifying beating frequency ((**F**), *n* = 4) and FP amplitude ((**G**), *n* = 4) in K_2_CO_3_-treated iPSC-CMs. Low doses (0.2–0.6 mM) increased frequency significantly, while 3.2 mM caused a strong decrease. FP amplitude was significantly reduced at 3.2 mM. All values are presented as mean ± SEM, * *p* < 0.05 vs. control (0 mM).

**Figure 5:**
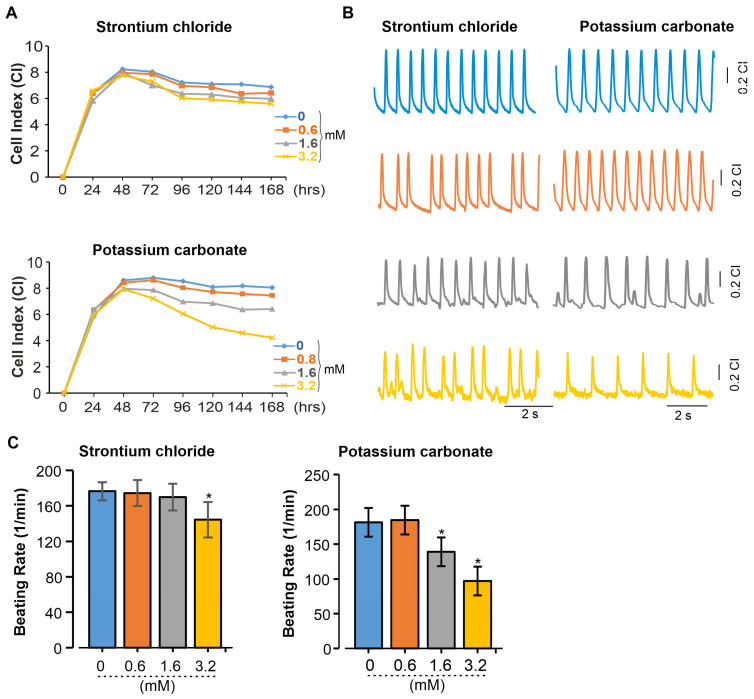
Functional effects of SrCl_2_ and K_2_CO_3_ on iPSC-CM were assessed using xCELLigence RTCA Cardio System. (**A**) A representative graph from one of three independent experiments showing the effects of SrCl_2_ and K_2_CO_3_ on miPSC-CMs. The CI was monitored in real time for about 196 h after the addition of 0 mM (control) or various concentrations of SrCl_2_ and K_2_CO_3_. (**B**) Representative beating rate pattern from 20 ms recordings of iPSC-CMs exposed to different concentrations of SrCl_2_ and K_2_CO_3_. (**C**) Average change in beating rate of iPSC-CMs under different concentrations of SrCl_2_ and K_2_CO_3_. Results are reported as the mean ± SEM (*n* = 3 independent experiments (6 wells each condition)). * *p* < 0.05 vs. control (0 mM).

To further investigate the effects of SrCl_2_ and K_2_CO_3_ on spontaneously beating clusters of cardiomyocytes, electrophysiological recordings were conducted using the MEA system. Figure 4A presents a representative image of day-12 embryoid bodies (EBs) plated onto an MEA chamber and recorded after attachment. As illustrated in Figure 5, low concentrations of both compounds had minimal impact on field potential amplitude and beating frequency (Figure 4B–G). Specifically, SrCl_2_ at a concentration range from 0.2 to 0.6 mM produced stable field potential traces without significant alterations in spike amplitude or frequency. However, at concentrations above 1.6 mM, SrCl_2_ induced noticeable changes in both parameters. In contrast, K_2_CO_3_ caused more pronounced and dose-dependent disruptions. At 3.2 mM, spike morphology became less defined, and field potential amplitude was reduced compared to the control. These effects were supported by quantitative analysis, which showed a consistent trend toward decreased spike amplitude at 1.6 mM and 3.2 mM for both compounds, although variations in beating frequency were less consistent. Taken together, both SrCl_2_ and K_2_CO_3_ exhibited minimal functional effects on iPSC-CMs at low concentrations, while higher concentrations led to significant electrophysiological disturbances, detectable through MEA-based field potential analysis. Field potential frequency and amplitude of spontaneous beating were used as primary endpoints to assess general disturbances in iPSC-CM activity. While these parameters allow detection of functional alterations, they do not provide a complete arrhythmogenic profile; metrics such as field potential duration, beat-to-beat variability, or conduction velocity were not assessed, and therefore, claims of arrhythmogenicity are presented cautiously.

### 2.4. Impedance-Based Analysis Reveals SrCl_2_ and K_2_CO_3_ Disrupt Coordinated Beating in iPSC-CMs

To distinguish the effects of SrCl_2_ and K_2_CO_3_ on the beating patterns of 2D cardiac cell cultures, real-time impedance analysis was performed using the xCELLigence RTCA system. This platform enables continuous, label-free monitoring of cellular dynamics, capturing changes in cell adhesion, morphology, and contractile activity. By analyzing impedance signals, alterations in beating frequency and potential arrhythmic events induced by chemical exposure or genetic modifications can be effectively assessed. As revealed, exposure of iPSC-CMs to SrCl_2_ and K_2_CO_3_ induced changes in the CI and beating pattern in a concentration-dependent manner (Figure 5A). Both compounds do not appear to impede cell attachment or monolayer formation, as indicated by the normal progression of the CI. At the high concentration of 3.2 mM, prolonged exposure led to a progressive decline in CI over 72 h for both compounds, with a more pronounced reduction observed for K_2_CO_3_. Lower concentrations (0.6–1.6 mM) had minimal effects on CI values compared to the controls. Beating profiles recorded via the RTCA system revealed that SrCl_2_ preserved rhythmic contractions at 0.6 and 1.6 mM. At 3.2 mM, contractions remained regular but showed reduced amplitude and frequency (Figure 5B, left). In contrast, K_2_CO_3_ at 3.2 mM induced irregular and low-amplitude beating (Figure 5B, right), indicating impaired excitation–contraction coupling. Quantification of beating frequency confirmed these observations (Figure 5C). SrCl2 caused a significant reduction only at 3.2 mM (*p* < 0.05), while K_2_CO_3_ induced a dose-dependent decrease starting at 1.6 mM, with frequencies dropping below 100 bpm at 3.2 mM (*p* < 0.05). These results demonstrate dose-dependent functional impairment in iPSC-CMs, with K_2_CO_3_ causing more severe and earlier effects than SrCl_2_.

### 2.5. Transcriptional Impact of SrCl_2_ and K_2_CO_3_ on iPSC-CMs

To assess the molecular impact of SrCl_2_ and K_2_CO_3_ on cardiac gene expression, quantitative reverse transcription polymerase chain reaction (qRT-PCR) analysis was performed at 24 h and 72 h following exposure to each compound at concentrations of 0.2 mM and 3.2 mM. The analysis focused on a set of genes representing key aspects of cardiac development and function, including early mesodermal differentiation (*Mesp1*), structural sarcomeric proteins (*Myl2*, *Myh6*, *Mlc2v*), cardiac ion channels (*Scn5a*, *Cacna1c*, *Kcnh2*), and a stress marker (*Nppa*). Expression levels were normalized to *Gapdh* as an internal reference.

**Figure 6:**
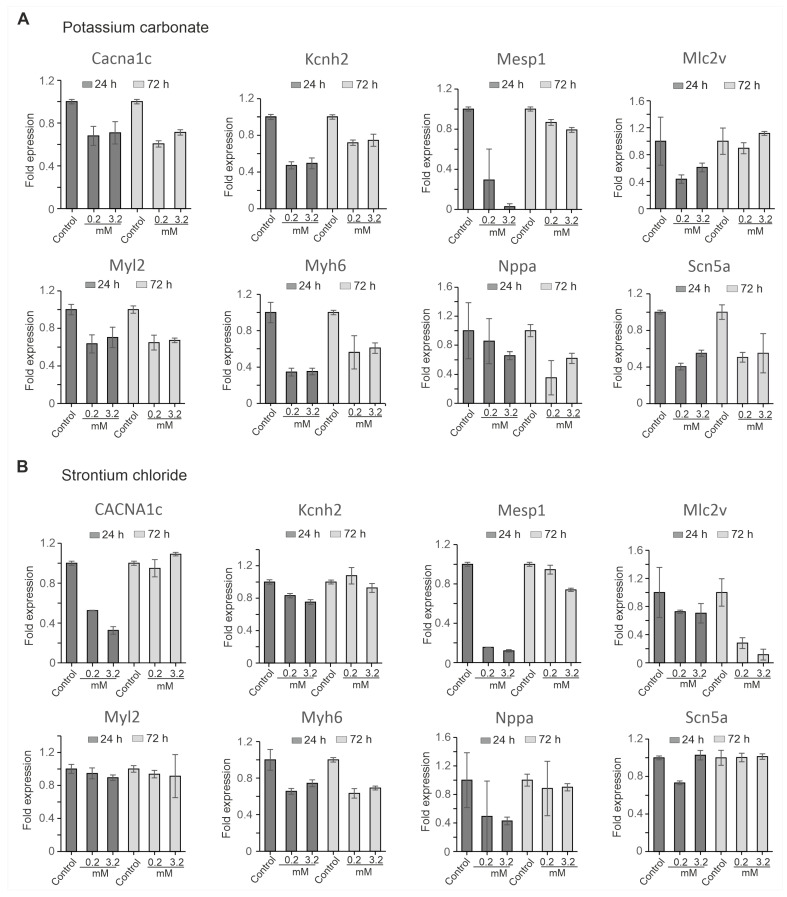
qRT-PCR analysis of cardiac gene expression in iPSC-CMs treated with SrCl_2_ and K_2_CO_3_. (**A**) Gene expression levels after treatment with 0.2 mM and 3.2 mM K_2_CO_3_ at 24 h and 72 h relative to the untreated control. Expression of cardiac progenitor marker (*Mesp1*), structural genes (*Myl2*, *Myh6*, *Mlc2v*), ion channel genes (*Scn5a*, *Cacna1c*, *Kcnh2*), and stress marker (*Nppa*) was assessed. K_2_CO_3_ exposure caused widespread and persistent downregulation across several markers, particularly *Mesp1*, *Myl2*, *Myh6*, and *Kcnh2*. (**B**) Gene expression levels following SrCl_2_ exposure under identical conditions. SrCl_2_ treatment induced more-transient changes, with several markers (*Mesp1*, *Cacna1c*, *Kcnh2*) showing partial or full recovery by 72 h. Data represent mean ± SEM from three independent experiments. Expression was normalized to *Gapdh* and calculated using the ΔΔCt method.

The result revealed that K_2_CO_3_ induced broad transcriptional suppression in a dose- and time-dependent manner (Figure 6A). *Mesp1* and *Myh6* were markedly downregulated at both time points, 24 h and 72 h, with the suppression being more pronounced at higher concentrations (3.2 mM) after 24 h. *Myl2* and *Kcnh2* showed persistent downregulation at all concentrations tested. While *Mlc2v* initially showed a decline, its expression recovered at 72 h, indicating some degree of compensation at the lower dose (0.2 mM). *Scn5a* and *Cacna1c* were also significantly affected, with *Scn5a* showing remarkable downregulation compared to *Cacna1*. In addition, *Nppa* progressively declined over time, with more pronounced suppression observed at the higher concentration. In contrast, SrCl_2_ elicited milder and more partially reversible changes in gene expression (Figure 6B). *Mesp1* and *Cacna1c* were transiently downregulated at 24 h in a dose-dependent manner, but both recovered by 72 h, indicating a temporary effect. Similarly, *Myl2* was moderately affected at both time points. *Mlc2v* and *Myh6* showed sustained downregulation at the tested concentration. *Scn5a* and were only slightly affected, and *Nppa* expression showed partial recovery after 72 h.

Taken together, K_2_CO_3_ caused more robust widespread transcriptional repression of multiple categories of genes, including markers of early mesodermal differentiation, structural sarcomeric proteins, ion channels, and stress markers. This indicates that K_2_CO_3_ has a higher transcriptional toxicity potential than SrCl_2_ in iPSC-CMs, which exhibited milder and more reversible changes in gene expression. These results suggest that K_2_CO_3_ may pose a greater risk to cardiac development and function than SrCl_2_, emphasizing the compound-specific nature of their gene expression profiles and their potential impact on cardiac cells.

## 3. Discussion

This study investigated the cytotoxic and cardiotoxic properties of two commonly used inorganic compounds, SrCl_2_ and K_2_CO_3,_ using a murine iPSC-derived in vitro model. By combining real-time impedance monitoring, electrophysiological recordings via MEA, FACS, and gene expression analysis, we assessed their impact on both undifferentiated iPSCs and differentiated iPSC-CMs. Our results demonstrate that both compounds interfere with CM viability and function in a dose- and time-dependent manner, although through distinct and potentially complementary mechanisms.

SrCl_2_ displayed a variable toxicity profile across assays. While some concentrations transiently stimulated iPSC proliferation, higher doses consistently reduced cell viability and altered contractility. These effects may be related to the chemical similarity between Sr^2+^ and Ca^2+^, which could allow Sr^2+^ entry via Ca^2+^ channels and interfere with Ca^2+^-dependent signaling pathways [14,15]. Previous studies revealed that Sr^2+^ can prolong action potentials (AP) and disrupt excitation–contraction coupling [16,17], consistent with the mild arrhythmogenic activity observed in our MEA and RTCA xCELLigence data. Gene expression analysis revealed transient suppression of differentiation and contractile markers, with partial recovery by 72 h, suggesting an adaptive cellular response or incomplete toxicity under the conditions tested. The variability in proliferation responses across replicates is consistent with reported cell-type-specific effects of SrCl_2_, which can promote proliferation in some contexts while being cytotoxic in others [18,19].

In contrast, K_2_CO_3_ exhibited a robust and consistent cytotoxic profile. Exposure to increasing concentrations led to a rapid decrease in cell index, pronounced morphological disruption, and high-density cell death. This is likely mediated by membrane depolarization due to excess extracellular K^+^ concentration, which may inactivate Na^+^ channels [20], and consequent impairment of AP initiation [21,22]. This observation is consistent with our MEA findings demonstrating reduced spike amplitude and contractile silencing. Additionally, the sustained downregulation of some cardiac genes, particularly encoding ion channels and structural proteins, suggests a broader disruption of CMs identity and functional integrity.

Both compounds also affected transcriptional programs and stress signaling. Downregulation of *Mesp1*, *Myh6*, and *Kcnh2* observed after compound exposure suggests disruption of cardiac identity, with K_2_CO_3_ inducing more consistent suppression. SrCl_2_ may interfere with transcription via Ca^2+^-mimetic signaling pathways, potentially implicating NFAT or MEF2 activation [23,24,25]. Persistent suppression of *Mlc2v* and transient *Nppa* modulation in SrCl_2_-treated cells may reflect early responses or alterations in ventricular cell and tissue specification. The observed downregulation of these genes may also reflect changes in cell composition, RNA integrity or viability rather than direct transcriptional repression, as differential expression in heterogeneous samples can be confounded by shifts in the proportions of cell types or cardiac subtypes [26,27]. It should also be noted that SrCl_2_ and K_2_CO_3_ might exert effects through mechanisms beyond electrophysiology. Since Sr^2+^ has been shown to activate the Ca^2+^-sensing receptor (CaSR), a G-protein coupled receptor involved in intracellular Ca^2+^ release via IP_3_ signaling in bone and thyroid cells [28,29], it remains plausible that a comparable mechanism may occur in CMs. In CMs, such dysregulation could trigger mitochondrial Ca^2+^ overload and apoptosis [30]. Similarly, excess extracellular K^+^ may modulate cytokine signaling and pro-apoptotic pathways [31], and carbonate ions of K_2_CO_3_ may locally alkalinize the medium, potentially introducing secondary stress [30]. Moreover, changes in pH and other ion gradients concurrently influence membrane channels and signaling [32]. Although not directly measured here, ionic imbalances can influence inflammatory pathways [33,34], including TGF-β and TNF-α signaling, which are known to affect CM survival and function [35,36]. We cannot exclude that variability in compound effects could reflect differences in CM subtype composition or maturation status in the heterogeneous stem cell-derived cardiac clusters [37].

In this study, the concentrations of K_2_CO_3_ and SrCl_2_ applied to iPSC-CMs were selected based on MEA dose–response analyses to represent two distinct functional outcomes: a low concentration with minimal observable effects and a higher concentration that produced pronounced alterations in spontaneous beating activity. We acknowledge that 3.2 mM of both compounds exceeds levels expected following normal human oral exposure and is therefore supraphysiological, representing a worst-case scenario. Such concentrations are commonly used in vitro to assess hazard potential and to explore concentration-dependent cellular responses, rather than to directly predict in vivo effects. Importantly, systemic exposure to toothpaste ingredients in humans is generally low, with only transient increases in salivary or oral-tissue concentrations, which are rapidly diluted and cleared [38,39,40].

In summary, SrCl_2_ and K_2_CO_3_ exhibit distinct, dose-dependent cardiotoxic profiles in iPSC-CMs, with Srl_2_ inducing progressive arrhythmogenic disturbances and K_2_CO_3_ causing acute electrical silencing. These findings highlight how even simple ionic compounds can exert distinct and complex effects on excitable cells. Although further validation is needed, our observations underscore the importance of functional cardiotoxicity assessments for commonly used substances, including those considered relatively inert. While our findings reveal dose-dependent cytotoxicity and functional alterations in murine iPSC-CMs, these results are based on acute in vitro exposures and should not be directly extrapolated to predict human safety, as in vivo metabolism, clearance, and dilution are not captured in this model.

### Limitations

This investigation offers initial insights into the acute effects of SrCl_2_ and K_2_CO_3_ on murine iPSC-CMs; however, several caveats must be acknowledged. The absence of direct mechanistic investigations, such as assessments of ion channel activity, Ca^2+^ handling, or inflammatory signaling, restricts the mechanistic interpretation of the observed cellular responses. Moreover, the concentrations applied in vitro were not benchmarked against physiologically relevant exposure levels, and corresponding pharmacokinetic or absorption data are unavailable to support translational extrapolation. The focus on short-term exposure (≤72 h) in both MEA recording and gene expression analyses further restricts the translation of these findings to realistic consumer exposure scenarios. Consequently, the present results should be considered hypothesis-generating and underscoring the need for follow-up studies that incorporate long-term, physiologically based, and mechanistically oriented approaches, including appropriate comparator substances and in vivo experiments, to more accurately delineate the cardiotoxic potential of these compounds.

## 4. Materials and Methods

### 4.1. Reagents and Compounds

All reagents were purchased from Carl Roth (Karlsruhe, Germany), unless otherwise stated. Dulbecco’s Modified Eagle Medium (DMEM, Thermo Fisher Scientific, Dreieich, Germany) was used to culture undifferentiated murine iPSCs, while Iscove’s Modified Dulbecco’s Medium (IMDM) served as the base for cardiac differentiation protocols. Stock solutions of SrCl_2_ and K_2_CO_3_ were freshly prepared and diluted in the respective culture media to achieve final concentrations ranging from 0.07 to 3.2 mM for SrCl_2_ and 0.1 to 3.2 mM for K_2_CO_3_, immediately prior to application.

### 4.2. Culture of Murine iPSCs

The murine αPig-AT25 iPSC line (CVCL_IS70), which expressed eGFP and puromycin resistance under control of the αMHC promoter as previously described [41], was maintained on mitotically inactivated mouse embryonic fibroblasts (MEFs). The cells were cultured in DMEM supplemented with 15% fetal bovine serum (FBS), 100 U/mL leukemia inhibitory factor (LIF), 1% non-essential amino acids, 1 mM sodium pyruvate, and 0.1 mM β-mercaptoethanol. Cells were passaged every 48 h using 0.05% trypsin/EDTA.

### 4.3. Cardiac Differentiation of iPSCs

Cardiomyocyte (CM) differentiation was induced using the embryoid body (EB) method with slight modifications based on Fatima et al. [41]. Briefly, 1 × 10^6^ iPSCs were cultured in suspension in IMDM supplemented with 20% FBS and 1% penicillin/streptomycin under continuous agitation. On day 2, the resulting EBs were plated onto gelatin-coated dishes and maintained under adherent conditions for an additional 7 days. Spontaneous contraction of the EBs was observed between days 7 and 9. Puromycin (8 µg/mL) was added for 72 h to select αMHC-positive CMs, resulting in the enrichment of purified iPSC-CMs.

### 4.4. Drug Treatments

To distinguish developmental versus functional cardiotoxicity, compound exposures were conducted either during EB formation/differentiation (assessing EB size, α-actinin staining, and viability) or on fully differentiated iPSC-CMs (assessing electrophysiology, impedance-based beating, and cardiac gene expression), with each stage analyzed as a separate experiment and including controls (untreated).

**Figure 7:**
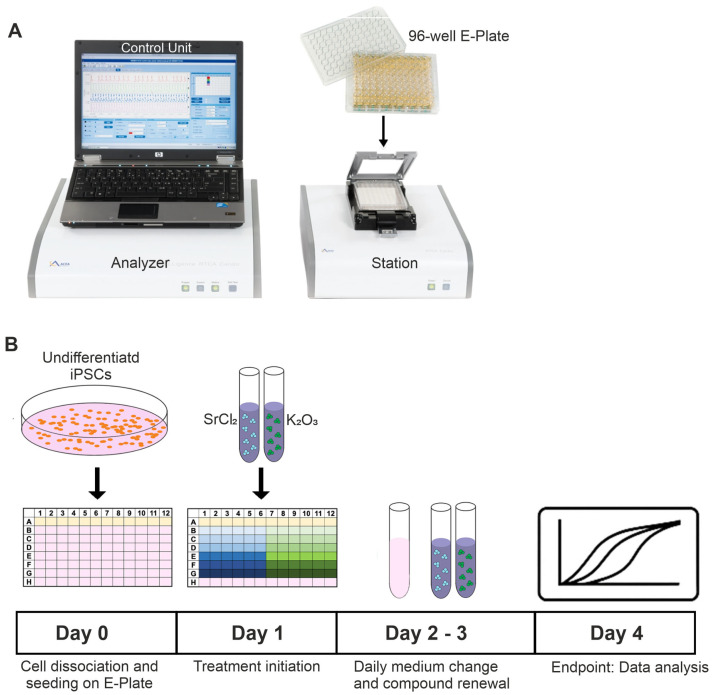
A schematic overview of the experimental setup for the xCELLigence-based proliferation monitoring of murine iPSCs. (**A**) The xCELLigence RTCA Cardio Instrument is used for continuous impedance-based cell proliferation and viability analysis. The system includes an analyzer and control unit, connected to specialized 96-well E-Plates containing interdigitated gold electrodes. (**B**) Timeline summarizing the experimental procedure: After seeding (day 0), treatment was initiated on day 1, and cells were exposed to repeated compound application for up to 72 h. Treatments with SrCl2 and K_2_CO_3_ were applied at various concentrations. Final analysis was performed on day 4.

For cytotoxicity and functional evaluations, cells were seeded in appropriate densities on gelatin-coated 96-well E-Plates (xCELLigence RTCA Cardio Instrument, Agilent Technologies, formerly ACEA Biosciences, Santa Clara, CA, USA, Figure 7A) or MEA chips and allowed to adhere for 24 h. After adhesion, the cells were then treated with various concentrations of SrCl_2_ or K_2_CO_3_. For the cell culture experiments, two concentrations (one low and one high) were selected based on the results of the MEA analysis. These concentrations represent experimental conditions at which either no functional effects (low) or marked functional effects (high) were observed. To maintain continuous exposure, the culture medium and compounds were refreshed every 24 h (Figure 7B).

### 4.5. xCELLigence Real-Time Cell Analysis (RTCA)

The proliferations and cardiotoxicity of iPSCs and iPSC-CMs were assessed using the xCELLigence RTCA Cardio Instrument (Figure 7A). Real-time changes in electrical impedance were recorded as cell index (CI), which reflects cell viability, adherence, and beating behavior (in case of iPSC-CMs) [42]. Data were collected and analyzed using RTCA Cardio Software 1.0.

### 4.6. Field Potential (FP) Recordings Using Microelectrode Arrays (MEA)

Extracellular FP recordings were conducted using a microelectrode array (MEA) recording system equipped with a Multichannel Systems 1060-Inv-BC amplifier and data acquisition system (Multichannel Systems, Reutlingen, Germany). Beating embryoid bodies (EBs) were cultured on MEA dishes that were pre-coated with 0.1% gelatin. The cultures were incubated at 37 °C in a 5% CO_2_ atmosphere for 1–2 days to allow proper attachment before recording. All recordings were carried out at 37 °C to maintain physiological conditions. Various concentrations of both SrCl_2_ and K_2_CO_3_ were applied to the MEA chambers containing the tissue cultures. After a 3 min baseline recording, different concentrations of both compounds (applied gradually) were introduced, with recordings taken for at least 3 min at each concentration. The data collected from the MEA were analyzed using custom-made LabView-based software (version 20, National Instrument, USA). The analysis focused on the characterization of field potential (FP) frequencies and amplitude.

### 4.7. Fluorescence-Activated Cell Sorting (FACS)

Cell viability and membrane integrity were assessed via propidium iodide (PI) staining. Following trypsinization, cells were washed with phosphate-buffered saline (PBS) and resuspended in PBS containing 1% fetal bovine serum (FBS). To stain dead cells, PI (1 µg/mL) was added immediately prior to analysis. Fluorescence was measured using a flow cytometer, and data were analyzed with FlowJo software (version 7). Both iPSCs and iPSC-CMs were assessed for cell death.

### 4.8. Quantitative Real-Time PCR (qRT-PCR)

Total RNA was extracted from iPSC-CMs using the RNeasy Mini Kit (Qiagen, Germany) according to the manufacturer’s instructions. cDNA synthesis was performed with High-Capacity cDNA Reverse Transcription Kit (Applied Biosystems, Carlsbad, CA, USA), using 1 µg of total RNA as starting material. Quantitative RT-PCR was carried out with SYBR Green Master Mix (Bio-Rad, Hercules, CA, USA) on a StepOnePlus^TM^ Real-Time PCR System (Applied Biosystems, USA). The expression levels of cardiac-specific markers, including *Myl2*, *Myh6*, *Mesp1*, *Nppa*, *Mlc2v*, *Scn5a*, *Cacna1c*, and *Kcnh2* were normalized to *Gapdh*. Data analysis was perform using the ΔΔCt method. The primer sequences are described in Table 1. All reactions were performed in technical triplicate with at least three biological replicates.

### 4.9. Statistical Analysis

Data are expressed as mean ± SD, averaged from at least three independent experiments or as a percentage relative to the control group (set to 100%). Statistical significance was assessed using Student’s *t*-tests for comparisons between two groups or one-way analysis of variance (ANOVA) for multiple-group comparisons, after confirming normality and equal variance of the data. Differences were considered statistically significant at *p* < 0.05.

## Figures and Tables

**Table 1:** List of primers used for quantitative RT-PCR. Sequences are shown from 5′ to 3′ direction.

Gene	Forward Primer	Reverse Primer
*Myh6*	AACAGGTGATGGCAAGATCC	GCTCAAAGTCAGCACCTTC
*Myl2*	AAAGAGGCTCCAGGTCCAAT	TCAGCCTTCAGTGACCCTTT
*Nppa*	GGGGGTAGGATTGACAGGAT	ACACACCACAAGGGCTTAGG
*Mesp1*	GTCTGCAGCGGGGTGTCGTG	CGGCGGCGTCCAGGTTTCTA
*Cacna1c*	AAGGCTACCTGGATTGGATCAC	GCCACGTTTTCGGTGTTGAC
*Kcnh2*	CGTGCTGCCTGAGTACAAGCT	TGTGAAGACAGCCGTGTAGATGA
*Scn5a*	GAAGAAGCTGGGCTCCAAGA	CATCGAAGGCCTGCTTGGTC
*Mlc2v*	AAAGAGGCTCCAGGTCCAAT	TCAGCCTTCAGTGACCCTTT
*Gapdh*	GGTGCTGAGTATGTCGTGGA	CGGAGATGATGACCCTTTTG

## Data Availability

The original contributions presented in this study are included in the article. Further inquiries can be directed to the corresponding author.

## Abbreviations

The following abbreviations are used in this manuscript:

K_2_CO_3_	Potassium carbonate
SrCl_2_	Strontium chloride
miPSCs	Mouse-induced pluripotent stem cells
iPSC-CMs	iPSC-derived cardiomyocytes
CMs	Cardiomyocytes
MEA	Multi-electrode array
EB	Embryoid body
FACS	Fluorescence-activated cell sorting
qRT-PCR	Quantitative reverse transcription polymerase chain reaction
CaSR	Ca^2+^-sensing receptor
